# MiR-217-5p inhibits smog (PM2.5)-induced inflammation and oxidative stress response of mouse lung tissues and macrophages through targeting STAT1

**DOI:** 10.18632/aging.204254

**Published:** 2022-08-29

**Authors:** Jianli Xie, Shaohua Li, Xiaoning Ma, Rongqin Li, Huiran Zhang, Jingwen Li, Xixin Yan

**Affiliations:** 1 Department of Rheumatic Immunology, The Third Hospital of Hebei Medical University, Shijiazhuang, Hebei, China; 2 Department of Respiratory Medicine, The First Hospital of Hebei Medical University, Shijiazhuang, Hebei, China; 3 Intensive Care Unit, Shijiazhuang People’s Hospital, Shijiazhuang, Hebei, China; 4 Office of Academic Research, The Second Hospital of Hebei Medical University, Shijiazhuang, Hebei, China; 5 College of Pharmacy, The Hebei Medical University, Shijiazhuang, Hebei, China; 6 Department of Respiratory Medicine, The Second Hospital of Hebei Medical University, Shijiazhuang, Hebei, China

**Keywords:** PM2.5, lung, stress response, miR-217-5p, inflammation

## Abstract

Objective: To explore the roles of macrophages’ miR-217-5p in the process of PM2.5 induced acute lung injury.

Methods: GEO database and KEGG pathway enrichment analysis as well as GSEA were used to predicted the miRNA and associated target signals. And then mice and RAW246.7 macrophages treated with PM2.5 to imitate PM2.5 induced acute lung injury environment and then transfected with miR-217-5p NC or miR-217-5p mimic. The levels of inflammatory factors TNF-α and anti-inflammatory factor IL-10 of mice serum were tested by ELISA. And the pathological changes and ROS level of mouse lung tissues were stained by HE and DHE staining. The proteins expression of phosphorylated-STAT1, total-STAT1, TNF-α, IFN-γ as well as p47, gp91, NOX4 in mice or RAW264.7 cells were tested by western blot or immunofluorescence of RAW264.7 cell slides.

Results: The results of bioinformatics analysis indicated the miR-217 as well as STAT1 were involved PM2.5 associated lung injury. After exposure to PM2.5, the decreased levels of serum TNF-α but not IL-10, consistent with reduced macrophages’ accumulation as well as decreased ROS levels in lung tissues in miR-217-5p mimic group vs miR-217-5p NC group mice, and moreover, the protein expression levels of phosphorylated--STAT1, total-STAT1, TNF-α, IFN-γ, p47, gp91 and NOX4 in mouse lung tissues and RTAW246.7 macrophages cells were all significantly reduced with miR-217-5p mimic administration. The above phenomena were reversed by specific STAT1-inhibitor HY-N8107.

Conclusions: miR-217-5p suppressed the activated STAT1-signal induced inflammation and oxidative stress trigged by PM2.5 in macrophages and resulted in the decreased lung injure caused by PM2.5.

## INTRODUCTION

Smog, as a product of modern social progress and development, has complex and diverse compositions that vary in different regions and seasons. Particulate matter 2.5 (PM2.5) is an important composition of smog, which seriously threatens human health. Long-term exposure to PM2.5 has a close association with respiratory diseases [1, 2]. The mechanism of damage of PM2.5 to the respiratory system primarily includes inflammatory response [3], oxidative stress response [4], immunotoxicity [5] and genotoxicity [6].

Acute lung injury is mostly caused by PM2.5, cellular characteristics include loss of alveolar-capillary membrane integrity, transepithelial migration of neutrophils and macrophages, and increases in proinflammatory/cytotoxic proteins, Persistent exposure of PM2.5 result in pulmonary destruction, fibrosing alveolitis and lung organism failure [7]. The immune system, especially the macrophages, represent as the susceptible and powerful defense mechanism against the harmful factors, PM2.5 included [8, 9], in normal conditions, alveolar macrophages will, through phagocytosis, immunity and secretion, immediately eliminate PM2.5, but when durative PM2.5 enter the respiratory system, the macrophages in lung become over-activated and bring about exacerbation of acute injury [10–12]. Activated macrophages secret pro-inflammatory cytokines (eg, TNFα, IFNs, interleukins), and generate cytotoxic reactive oxygen species (ROS) and proteolytic enzymes, and bioactive lipids, and moreover, extracellular traps (fibers composed of DNA and proteins) released from inflammatory macrophages in response to ROS, proteases, and TNFα in the pathogenic response to pulmonary toxicants deteriorate the acute lung injury [7, 13]. The generation of these ROS and inflammatory proteins are controlled by several primary signaling pathways, such as NF-κB, AP-1, STATs, and epigenetic regulators (eg, microRNAs, chromatin modifiers) that are activated and participate in the post-translational regulatory networks [7, 14].

More interestingly, PM2.5 can alter the expression of micro ribonucleic acids (miRNAs) in the body [15]. In the present study, a differentially expressed gene miR-217-5p was found through GEO database and bioinformatics analysis, and the JAK-STAT signaling pathways were enriched pathways. Signal transducer and activator of transcription 1 (STAT1) is phosphorylated in the cytoplasm, and dimer is formed, followed by nuclear translocation, which acts on the promoter of target genes such as ROS, thus promoting M1 polarization of macrophages and amplifying inflammatory responses. Therefore, enrichment analysis and pathway analysis were conducted from bioinformatics strongly pointed out the involvement and relationship of miR-217-5p, PM2.5 -induced inflammation and oxidative stress of macrophages, therefore, our study may provide potentially valuable biomarkers or mechanisms for early warning of lung injury diseases caused by air pollution.

## RESULTS

### Screening of DEGs

**Figure 1:**
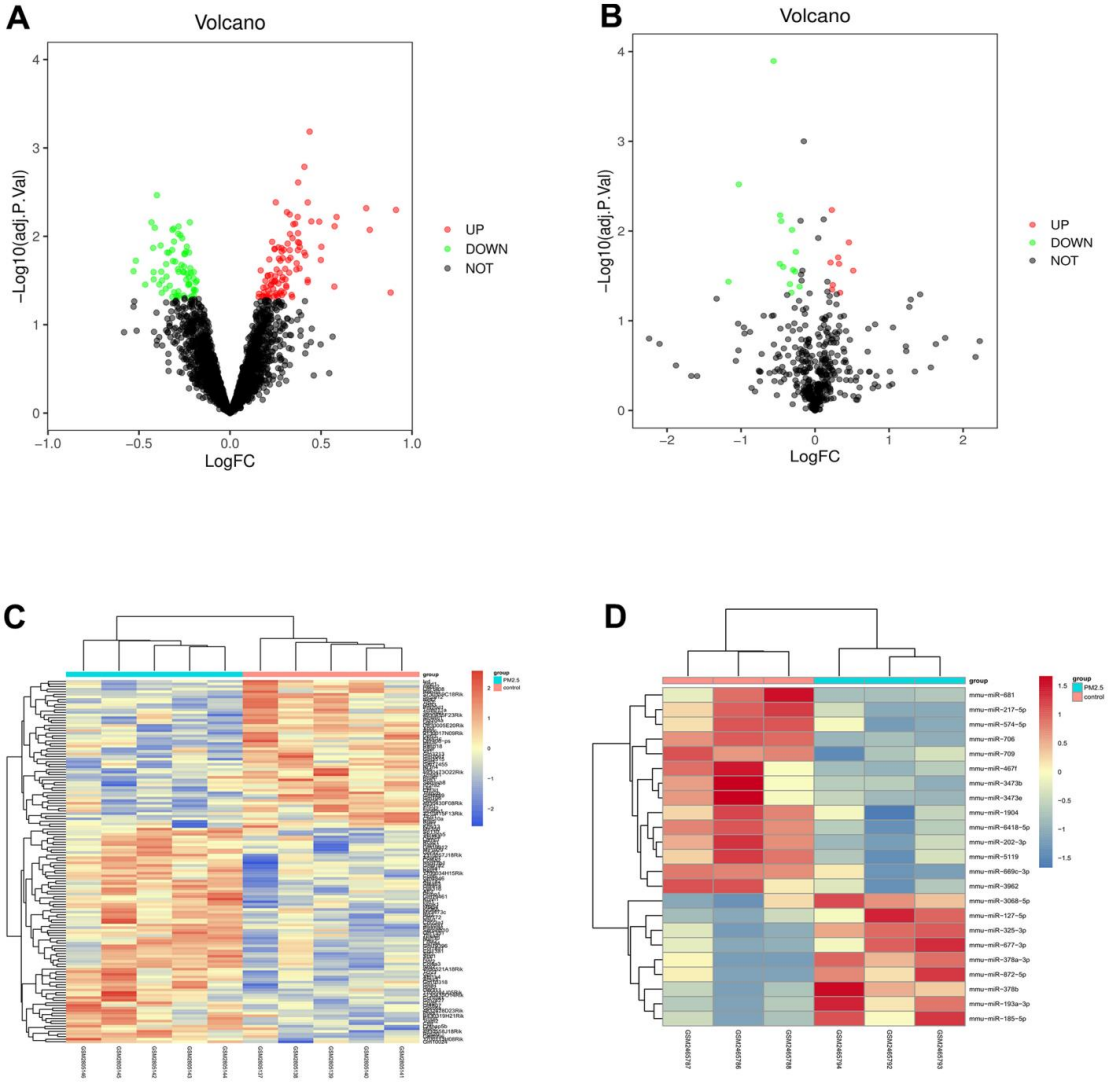
**Screening of differential gene (DEG).** (**A**, **B**) showed the volcano plot of visual grouping of DEGs in the dataset GSE104656 and GSE62819. (**C**, **D**) represented the cluster analysis heat map of DEGs in the dataset GSE104656 and GSE62819.

The dataset GSE104656 related to lung injury was downloaded from the GEO database, and the data were subjected to quantile normalization, followed by screening in GSE104656 (*p*<0.05 and |logFC|<1). The results showed that there were 145 DEGs in the mRNAs of lung injury, including 85 up-regulated ones and 60 down-regulated ones. The volcano plot of visual grouping of DEGs in the dataset GSE104656 was constructed using the ggplot2 package of R software (Figure 1A), and the cluster analysis heat map of DEGs was plotted using the pheatmap package of R software (Figure 1B). In addition, the dataset GSE62819 was subjected to quantile normalization, the DEGs were screened, and the volcano plot of DEGs (Figure 1C) and its cluster analysis heat map (Figure 1D) were constructed in the same way.

### Bioinformatics analysis

**Figure 2:**
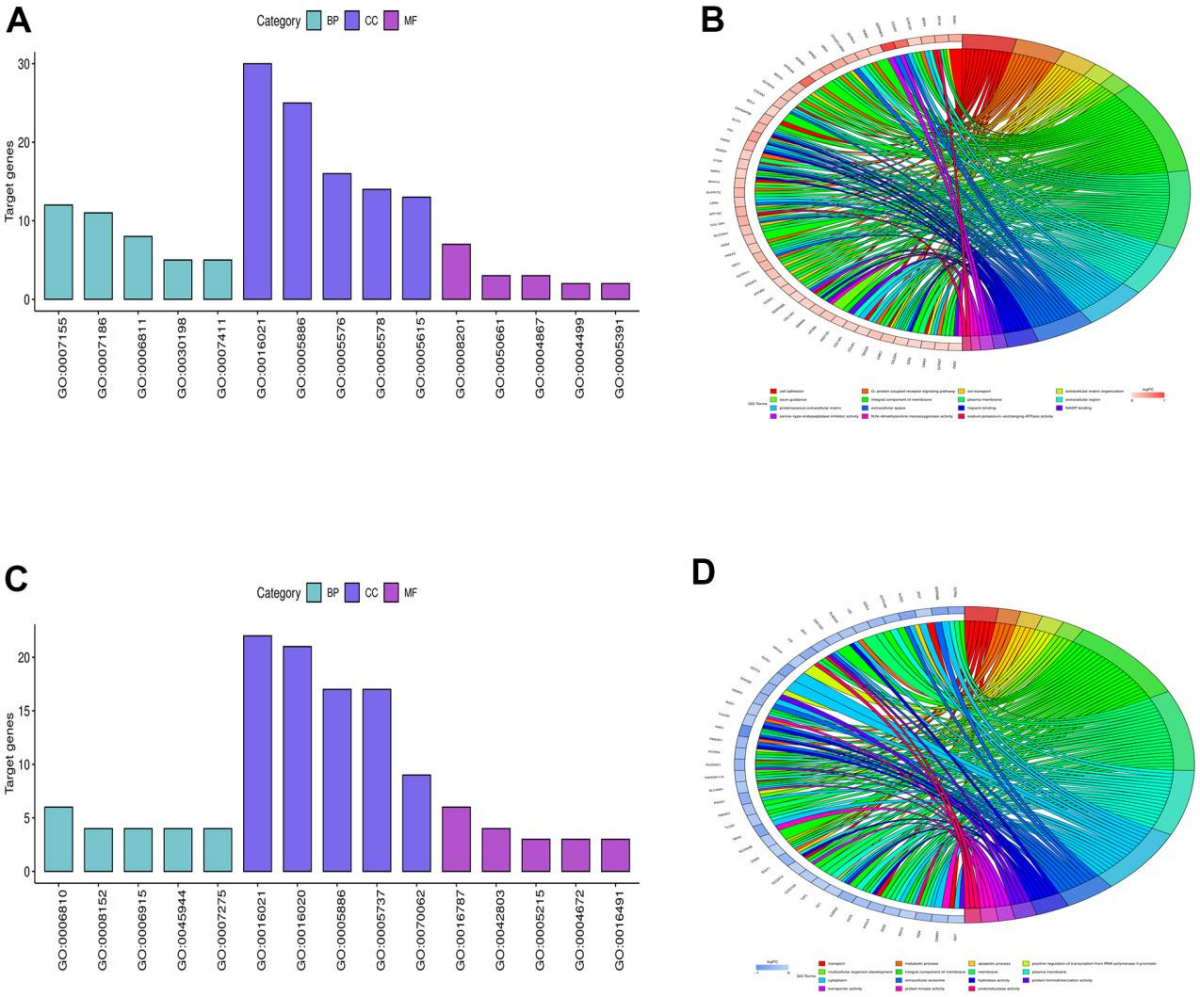
**Bioinformatics analysis.** (**A**, **B**) showed the diagrams of up-regulated GO pathways, and (**C**, **D**) represented the diagrams of down-regulated pathways of DEGs.

The DEGs obtained from GSE104656 were analyzed by GO and KEGG enrichment analyses. The DEGs at the biological process level were analyzed using the online database tool DAVID (https://david.ncifcrf.gov/) to integrate the GO terms, and the biological process network of DEGs was created. Moreover, the diagrams of up-regulated GO pathways (Figure 2A, 2B) and down-regulated pathways of DEGs (Figure 2C, 2D) were plotted using the R language. The up-regulated pathways included cell adhesion and extracellular matrix organization, and the down-regulated pathways included transport, metabolic process and apoptotic process, which were the enriched pathways of lung injury.

### Prediction of miRNA target genes

**Figure 3:**
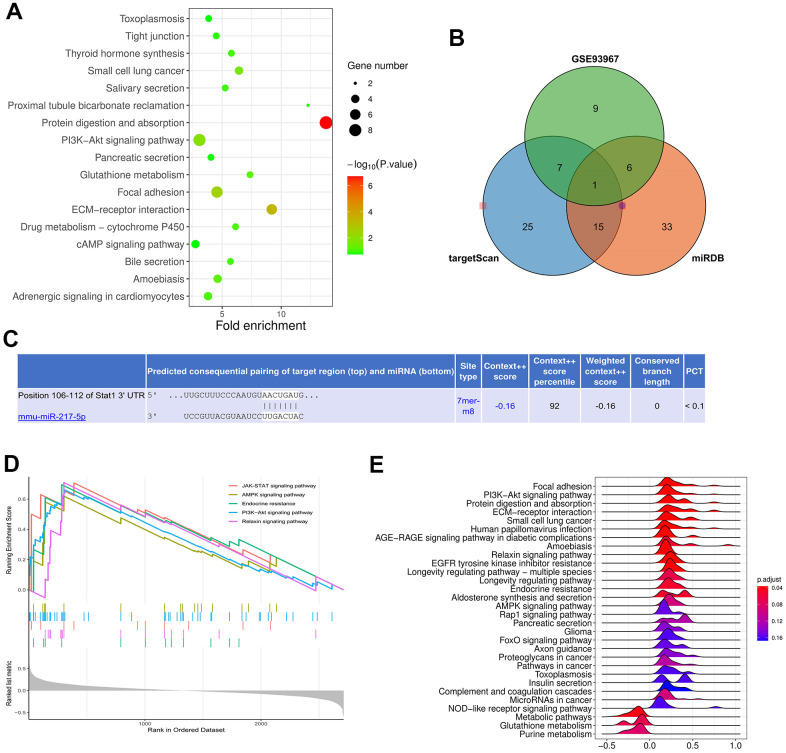
**Prediction of miRNA target genes.** (**A**) showed the KEGG pathway diagram. (**B**, **C**) represented Venn diagram and binding sites of mRNA and miRNA. (**D**, **E**) showed that JAK-STAT and AMPK signaling pathways were enriched pathways.

The DEGs were subjected to KEGG enrichment analysis, and the KEGG pathway diagram was plotted (Figure 3A). The candidate miRNA target genes were predicted using the online tools TargetScan and mirDIP. They together with DEGs in GSE62819 were plotted into Venn diagram using the VennDiagram package, and the intersection was taken. Then miR-217 binding to all up-regulated miRNAs was found (Figure 3B). Moreover, the binding sites between mRNA and miRNA were plotted based on the prediction results (Figure 3C). It was found by GSEA that the JAK-STAT and AMPK signaling pathways were enriched pathways (Figure 3D, 3E).

### Changes in the content of serum TNF-α, IFN-γ and IL-10

**Figure 4:**
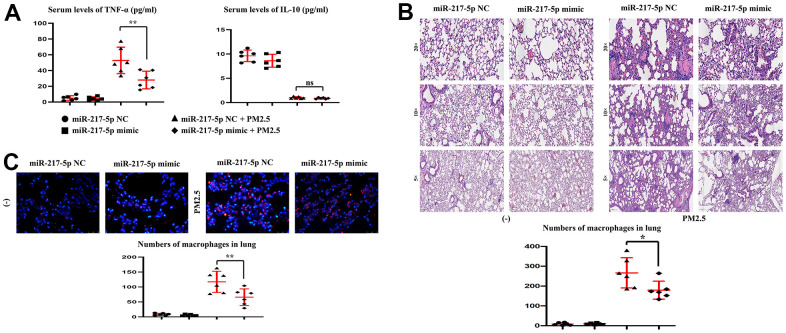
**Changes in the content of serum TNF-α and IL-10, and Lung histopathology.** (**A**) showed changes in the content of serum TNF-α and IL-10. (**B**, **C**) represented lung histopathology and numbers of macrophages in lung.

After treatment with PM2.5, the content of serum pro-inflammatory factors, TNF-α and IFN-γ, was significantly increased, while that of anti-inflammatory factor IL-10 was significantly decreased. After the exogenous overexpression of miR-217-5p, the content of serum TNF-α and IFN-γ in miR-217-5p mimic+PM2.5 group was significantly lower than that in miR-217-5p NC+PM2.5 group, and there was no obvious change in IL-10 (Figure 4A).

### Lung histopathology

Mice treated with clean air had normal lung tissues, without congestion and edema in miR-217-5p NC group and mimic group. After treatment with PM2.5, the alveolar space became narrow, the alveoli were locally ruptured, the alveolar septum became widened, and massive inflammatory cell infiltration was found in miR-217-5p NC+PM2.5 group compared with those in miR-217-5p NC group and miR-217-5p mimic group. In miR-217-5p mimic+PM2 group, the lung tissue injury was mild, the inflammatory cell infiltration was alleviated, and the pulmonary capillary had intact structure, but a little bleeding could still be seen and the numbers of lung macrophages in 20×field were shown in Figure 4B. The results of immunofluorescence staining revealed that the number of macrophages in the lungs was extremely small in miR-217-5p NC group and miR-217-5p mimic group. After treatment with PM2.5, a large number of macrophages in the lungs aggregated, but the number of macrophages in the lungs significantly declined after treatment with miR-217-5p mimic.

### Changes in reactive oxygen species (ROS) level in lung tissues

**Figure 5:**
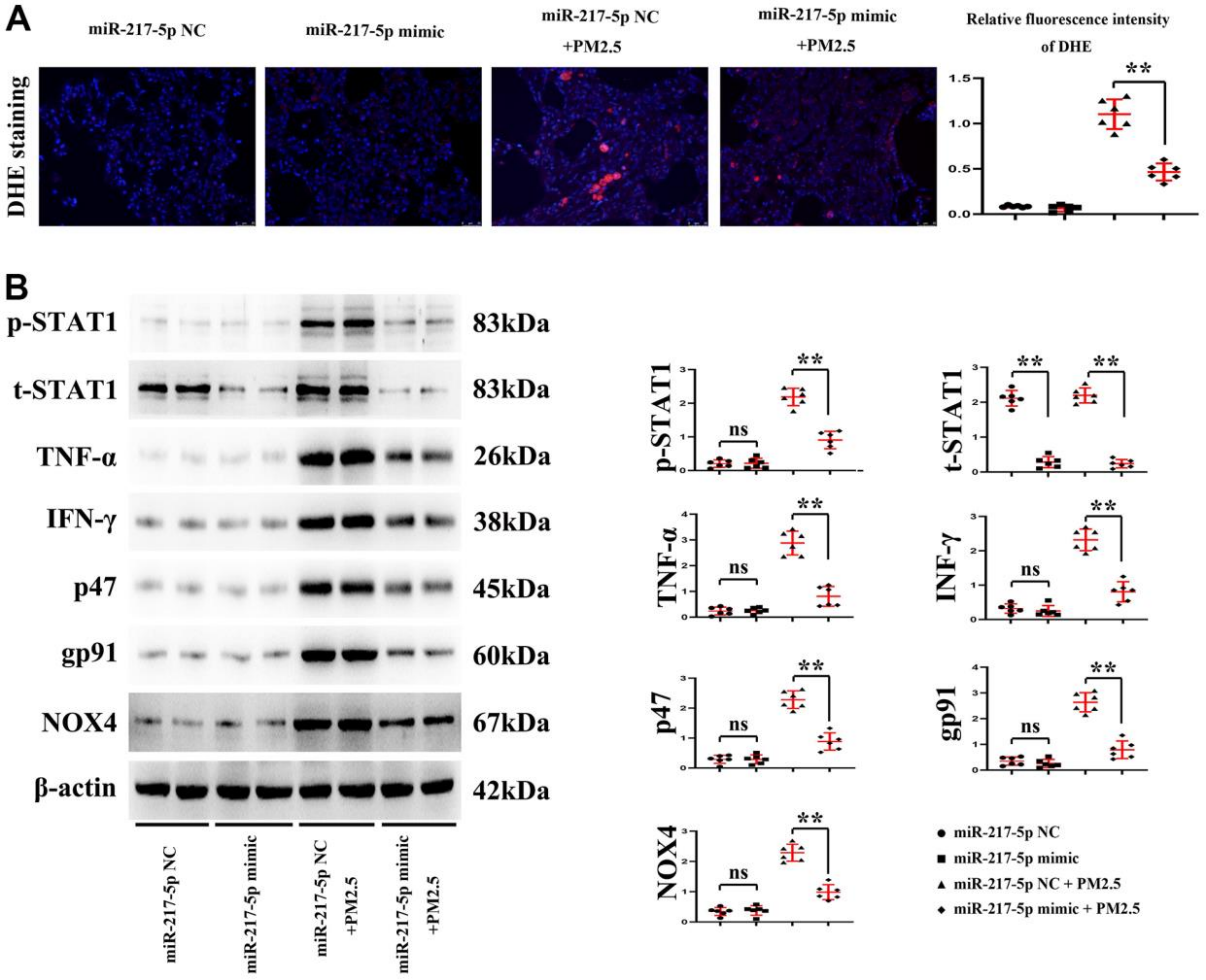
**Changes in reactive oxygen species (ROS) level in lung tissues.** (**A**) showed fluorescence intensity of DHE, and (**B**) represented the protein expressions of p-STAT1, t-STAT1, TNF-α, IFN-γ, p47, gp91 and NOX4 in lung tissues.

As can be seen from DHE staining, the red fluorescence intensity in the lung tissues was significantly higher in mice treated with PM2.5 than that of mice untreated with PM2.5. Compared with that in miR-217-5p NC+PM2.5 group, the red fluorescence intensity in the lung tissues significantly declined in miR-217-5p mimic+PM2.5 group (Figure 5A). The above findings indicate that PM2.5 can raise the ROS level in lung tissues of mice, while miR-217-5p mimic can lower the ROS level in lung tissues of mice after treatment with PM2.5. And the statistical outputs of these proteins tested by western blot were shown in Table 1.

**Table 1:** Statistical output of index of lung tissues tested by western blot.

**Index**	**Groups**	**NC**	**mimic**	**NC+PM2.5**	**mimic+PM2.5**
p-stat1	χ±s	0.203±0.111	0.213±0.147	2.187±0.258	0.904±0.259
	T value	0.129		8.606	
	P value	0.9		<0.001	
t-stat1	χ±s	2.113±0.222	0.283±0.159	2.195±0.213	0.238±0.119
	T value	16.4		19.63	
	P value	<0.0001		<0.0001	
TNF-α	χ±s	0.236±0.153	0.255±0.112	2.88±0.462	0.808±0.385
	T value	0.241		8.436	
	P value	0.814		<0.0001	
IFN-γ	χ±s	0.317±0.136	0.202±0.058	2.319±0.314	0.806±0.292
	T value	1.911		8.628	
	P value	0.0851		<0.0001	
p47	χ±s	0.289±0.135	0.288±0.149	2.282±0.293	0.884±0.289
	T value	0.007		8.313	
	P value	0.9946		<0.0001	
pg-91	χ±s	0.347±0.158	0.256±0.157	2.636±0.372	0.789±0.341
	T value	0.995		8.961	
	P value	0.343		<0.0001	
NOX4	χ±s	0.345±0.131	0.379±0.157	2.288±0.279	0.983±0.247
	T value	0.405		8.578	
	P value	0.694		<0.0001	

### Expression of p-STAT1 in RAW246.7 cells

**Figure 6:**
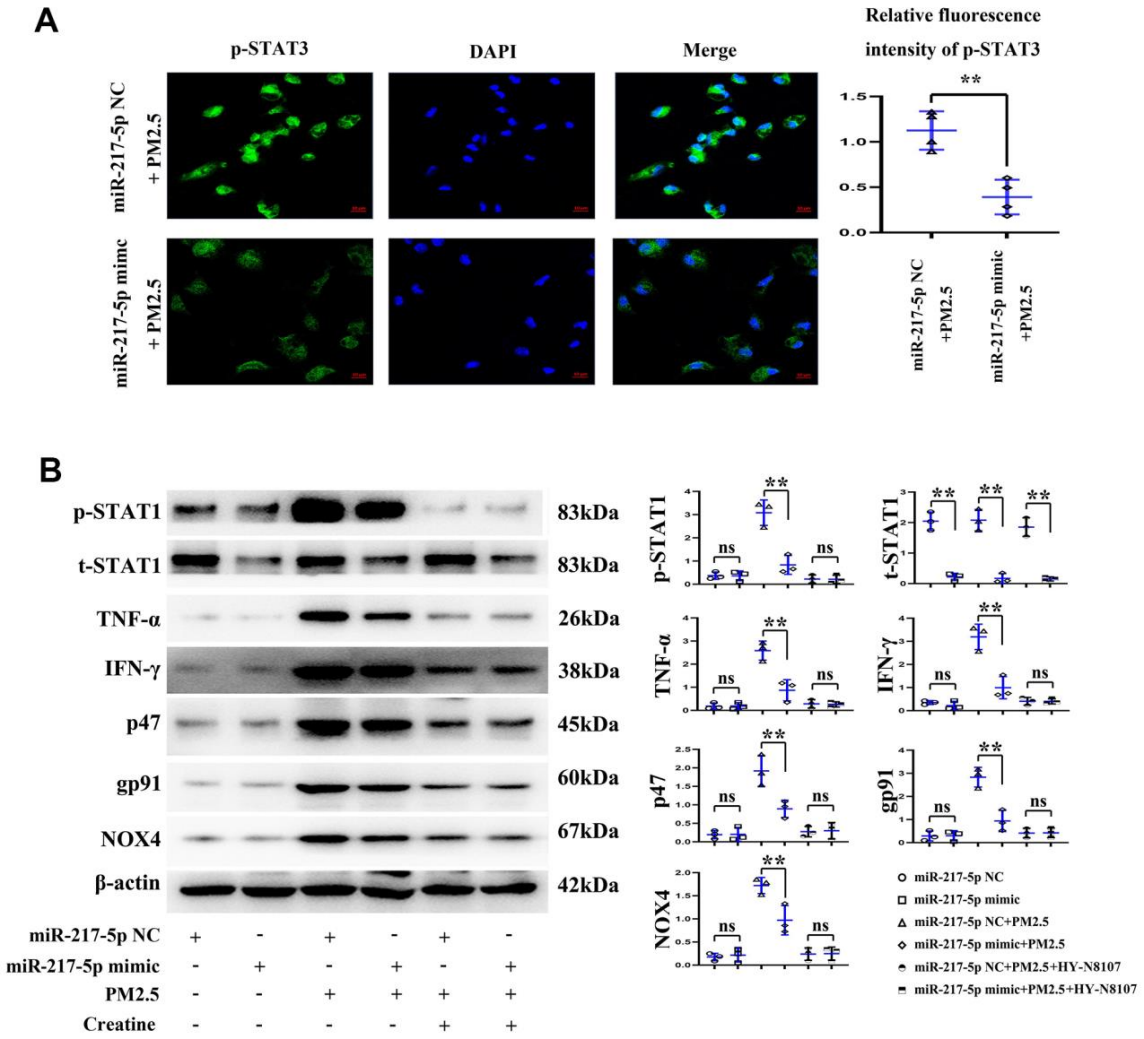
**Changes in reactive oxygen species (ROS) level in RAW246.7 cells.** (**A**) showed fluorescence intensity of p-STAT1, and (**B**) represented the protein expressions of p-STAT1, t-STAT1, TNF-α, IFN-γ, p47, gp91 and NOX4 in RAW246.7 cells treated with miR-217-5p mimic, PM2.5 and creatine.

The results of immunofluorescence of cell slides showed that the fluorescence intensity of RAW246.7 cells in miR-217-5p mimic+PM2.5 group was significantly lower than that in miR-217-5p NC+PM2.5 group (Figure 6A), suggesting that miR-217- 5p mimic can reduce the expression of p-STAT1 in PM2.5-contaminated RAW246.7 cells, and statistical output of p-STAT1 in RAW264.7 cells tested by western blot was shown in Table 2.

**Table 2:** Statistical output of index of RAW264.7 cells tested by western blot.

**Index**	**Groups**	**NC**	**mimic**	**NC+PM2.5**	**mimic+PM2.5**	**NC+PM2.5+Creatine**	**mimic+PM2.5+Creatine**
p-stat1	χ±s	0.368±0.156	0.361±0.210	3.082±0.54	0.831±0.40	0.221±0.177	0.211±0.180
	T value	0.05		5.72		0.07	
	P value	0.97		0.00		0.95	
t-stat1	χ±s	2.045±0.304	0.217±0.119	2.079±0.351	0.169±0.156	1.851±0.305	0.150±0.067
	T value	9.69		8.59		9.41	
	P value	0.00		0.00		0.00	
TNF-α	χ±s	0.182±0.138	0.184±0.146	2.58±0.41	0.867±0.45	0.280±0.183	0.255±0.114
	T value	0.02		4.82		0.20	
	P value	0.99		0.01		0.85	
IFN-γ	χ±s	0.347±0.084	0.219±0.182	3.19±0.547	0.992±0.48	0.403±0.168	0.402±0.113
	T value	1.11		5.24		0.01	
	P value	0.33		0.01		0.99	
p47	χ±s	0.192±0.113	0.200±0.185	1.918±0.41	0.891±0.23	0.273±0.140	0.299±0.221
	T value	0.06		3.72		0.18	
	P value	0.95		0.02		0.87	
pg-91	χ±s	0.288±0.218	0.298±0.219	2.83±0.425	0.936±0.45	0.411±0.206	0.422±0.209
	T value	0.06		0.01		0.07	
	P value	0.96		5.25		0.95	
NOX4	χ±s	0.173±0.080	0.213±0.148	1.72±0.174	0.970±0.317	0.237±0.134	0.247±0.141
	T value	0.41		3.59		0.08	
	P value	0.70		0.02		0.94	

### Protein expressions of p-STAT1, t-STAT1, TNF-α, IFN-γ, p47, gp91 and NOX4 in mouse lung tissues and RAW246.7 cells

According to the results of Western blotting, the protein expression of t-STAT1 in mouse lung tissues and RAW246.7 cells significantly declined in miR-217-5p mimic compared with those in miR-217-5p NC group, while other proteins had no obvious changes. After treatment with PM2.5, the protein expressions of p-STAT1, t-STAT1, TNF-α, IFN-γ, p47, gp91 and NOX4 significantly rose in mouse lung tissues and RAW246.7 cells and under the treatment with creatine, these inflammatory and ROS factors were significant suppressed and these results were shown in Figure 6B. The protein expressions in mouse lung tissues and RAW246.7 cells all significantly declined in miR-217-5p mimic+PM2.5 group compared with those in miR-217-5p NC+PM2.5 group. and moreover, the statistical outputs of these inflammatory factors and ROS in RAW264.7 cells tested by western blot were shown in Table 2.

## DISCUSSION

As an important index of air quality monitoring, PM2.5 has direct and severe damage to the human respiratory system [16], in this study, we hypothesized the relationship of PM2.5, over-activated macrophages, and regulatory molecules miR-217-5p in acute lung injury, and we elaborated the PM2.5 activated miR-217-5p modulated STAT1 signals in lung macrophages and --therefore amplified the burst of inflammatory response as well as ROS. The innate and adaptive immune system are strong involvement in the PM2.5 induced acute lung injury (ALI), especially the macrophages, which are major sources for inflammatory and ROS responses, and moreover the JAK/STAT1 signals are significant modulators for this stress and accelerate the initiation and amplification of pulmonary process of ALI caused by PM2.5 [17, 18]**.** The miRNAs are important molecules of gene or transduction signals’ regulation, whose pathogenic mechanism has been well clarified in many diseases [19]. It has been shown that miRNAs are implicated in air pollution-induced respiratory diseases and associated with decline in lung function [20]. However, the specific mechanism remains to be further studied. In the present study, therefore, the DEGs were screened from the GEO database, the candidate miRNA target genes were predicted using miRDB and TargetScan, and they together with DEGs in GSE62819 were plotted into Venn diagram using the VennDiagram package. The intersection was taken, and then miR-217 binding to all up-regulated miRNAs was found. Moreover, the binding sites between mRNA and miRNA were plotted based on the prediction results. Finally, it was found by GSEA that the JAK-STAT and AMPK signaling pathways were enriched pathways.

It was pathologically observed that after PM2.5 contamination, the alveolar space became narrow, the alveoli were locally ruptured, the alveolar septum became widened, and massive inflammatory cell infiltration was found. In mice injected with miR-217-5p mimic, the lung tissue injury was mild, the inflammatory cell infiltration was alleviated, and the pulmonary capillary had intact structure, but a little bleeding could still be seen. It can be seen that miR-217-5p can inhibit PM2.5-induced pathological damage to lung tissues of mice. At the same time, the content of serum TNF-α significantly increased but not IL-10 levels in miR-217-5p NC+PM2.5 group. TNF-α is a pro-inflammatory cytokine mainly produced by activated mononuclear macrophages at the initial stage of the inflammatory response. Both TNF-α and IFN-γ are more representative pro-inflammatory factors, and they can enhance aggregation of various inflammatory mediators and inflammatory cells, based on which the immune response is related to inflammatory response and tissue damage. Therefore, the content of serum TNF-α in miR-217-5p mimic+PM2.5 group were significantly lower than those in miR-217-5p NC+PM2.5 group, suggesting that miR-217-5p can inhibit PM2.5-induced secretion of pro-inflammatory factors in mice. IL-10 produced by immune cells such as lymphocytes and macrophages possesses a potent anti-inflammatory effect. After PM2.5 contamination, the level of serum IL-10 significantly declined in mice, indicating that PM2.5 reduces the secretion of anti-inflammatory factors in mice, so that the immune resistance to external factors may be weakened. Then it was found by DHE staining of frozen sections that miR-217-5p mimic could lower the level of ROS in lung tissues of PM2.5-treated mice. Under normal conditions, there is oxidation-antioxidant balance in the body, but the ability of cells to produce ROS can be greatly enhanced by various stress factors (such as endotoxin) to break the oxidation-antioxidant balance, thus leading to oxidative damage [21].

**Figure 7:**
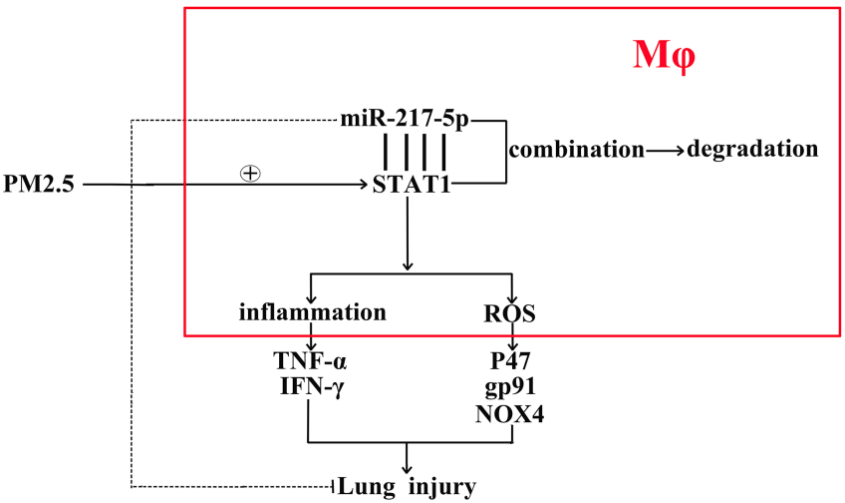
The schematic diagram of the miR-217-5p’ roles in the acute lung injury caused by PM2.5.

In this study, the expression levels of proteins associated with inflammation and oxidative stress in lung tissues and RAW246.7 cells were further detected *in vivo* and *in vitro*. Compared with that in miR-217-5p mimic group and miR-217-5p NC group, the protein expression of t-STAT1 in lung tissues and RAW246.7 cells significantly declined, suggesting that miR-217-5p can inhibit the total protein expression of STAT1. After treatment with PM2.5, the expressions of both p-STAT1 and t-STAT1 were significantly increased in lung tissues and RAW246.7 cells, but they were significantly lower in miR-217-5p mimic+PM2.5 group than those in miR-217-5p NC+PM2.5 group. MiR-217-5p could not only reduce the total protein expression of STAT1, but also inhibit the phosphorylation of STAT1. STAT1 is phosphorylated in the cytoplasm, and the dimer is formed, followed by nuclear translocation, which acts on the promoter of target genes such as iNOS, thus promoting M1 polarization of macrophages and inducing inflammatory response, thereby, the Creatine, a specific inhibitor of JAK/STAT1, were used in cellular experiments and identified the STAT1’ effects on inflammatory and ROS responses. MiR-217-5p also suppressed the PM2.5-induced up-regulation of TNF-α and IFN-γ protein expressions. Besides, the massive production of ROS can lead to the peroxidation of proteins, lipids and nucleic acids, ultimately resulting in cell injury and even death [22]. NOX is an important enzyme for the production of ROS, and inhibiting its production can relieve oxidative stress [23]. It has been shown that NOX4 constitutively catalyzes the production of ROS, and the activity of NOX4 is regulated at the transcriptional level [24]. Meanwhile, p47 and gp91 are also necessary for NOX activation [25]. MiR-217-5p mimic suppressed PM2.5-induced protein expression levels of p47, gp91 and NOX4, thereby alleviating the PM2.5-induced oxidative stress. In conclusion, overexpression of miR-217-5p inhibits PM2.5-induced inflammation and oxidative stress of mouse lung tissues and RTAW246.7 macrophages, and moreover, the mechanisms of miR-217-5p in the progression of PM2.5-induced lung inflammation were showed as a schematic diagram in Figure 7.

## MATERIALS AND METHODS

### Bioinformatics analysis

The datasets related to PM2.5-associated lung injury were searched from the Gene Expression Omnibus (GEO) database (https://www.ncbi.nlm.nih.gov/gds/). The dataset GSE104656 of lung injury-related messenger RNA (mRNA) expression was found and downloaded, and the dataset GSE62819 containing miRNA sequencing data of lung injury patients was also downloaded. The RNA-seq data were subjected to quantile normalization and the differentially expressed genes (DEGs) were analyzed using the Limma package of R language (|logFC|<1, *p*<0.05). Next, the volcano plot of visual grouping of DEGs in the dataset GSE104656 was constructed using the ggplot2 package of R software, and the cluster analysis heat map of DEGs was plotted using the pheatmap package of R software. In addition, the volcano plot of visual grouping of DEGs in the dataset GSE62819 and its cluster analysis heat map were constructed in the same way.

### Functional enrichment analysis

The DEGs in the dataset GSE104656 were subjected to Gene Ontology (GO) and Kyoto Encyclopedia of Genes and Genomes (KEGG) enrichment analyses. The DEGs at the biological process, cellular component and molecular function levels were analyzed using the online database tool DAVID (https://david.ncifcrf.gov) to integrate the GO terms, and the biological process network of DEGs was also created. The GO and KEGG pathway enrichment analysis diagrams of DEGs were plotted using the GOplot and ggplot2 packages of R software.

### Gene set enrichment analysis (GSEA)

GSEA was conducted on all genes using the GSEA tool (http://www.gsea-msigdb.org/), and the GSEA pathway enrichment analysis diagram was also plotted.

### Prediction of miRNA target genes

The candidate miRNA target genes were predicted using the online tools miRDB and TargetScan, and they together with DEGs in GSE62819 were plotted into Venn diagram using the VennDiagram package to predict the target genes. Moreover, the binding sites between mRNAs and miRNAs were plotted based on the prediction results.

### Modeling and grouping of mice

The male C57/BL6j mice aged 6-8 weeks were purchased from Skbex Biotechnology Co., Ltd., and the mice were randomly divided into 4 groups, n=6/group. MiR-217-5p mimic was injected via the caudal vein to interfere with the expression of miR-217-5p in mice, and miR-217-5p negative control (NC) was injected via the caudal vein as a NC. The animals were divided into miR-217-5p NC group, miR-217-5p mimic group, miR-217-5p NC+PM2.5 group and miR-217-5p mimic+PM2.5 group.

### Preparation of PM2.5

The samples were continuously taken using a medium volume sampler (TH150F, Wuhan Tianhong Instruments Co., Ltd.) and a quartz filter membrane (diameter =80 mm) at a flow rate of 100 L/min for 24 h/d in Shijiazhuang, Hebei Province. Then the quartz filter membrane adsorbing PM2.5 was cut into 4 pieces and put in a beaker filled with ultrapure water, and the mouth of the beaker was wrapped with tin foil, followed by ultrasonic oscillation for 30 min. The eluted PM2.5 was put into a refrigerator at -80° C after cooling to room temperature. After vacuum freeze-drying for 24 h, the sample eluent was irradiated with ultraviolet for 1 h and added with sterile water to be prepared into PM2.5 sample stock solution. After autoclaving, the solution could be used for cell experiments, and it should be shaken evenly before use.

### Cell culture and transfection

RAW264.7 macrophages purchased from Shanghai Institute of Biochemistry and Cell Biology, Chinese Academy of Sciences were cultured in Dulbecco's modified Eagle medium (DMEM, Gibco BRL) containing 10% fetal bovine serum, 100 mmol/L penicillin and 100 mmol/L streptomycin in a 5% CO_2_ incubator at 37° C, and passaged every 1-2 d. The cells in the logarithmic growth phase were harvested for experiments. The RAW264.7 cells were transfected with miR-217-5p mimic: miR-217-5p mimic, 5’-UAC UGC AUC AGG AAC UGA UUG GA-3’; miR-NC mimic, 5’-UUG UCC GAA CGU GUC ACG U-3’ (Shanghai Genechem Co. Ltd.) and stimulated with or without Creatine (10 μM, Selleck) respectively, and those in the logarithmic growth phase were digested and counted. After the concentration was adjusted, the suspension was inoculated into a 6-well plate (5×10^5^ cells/mL), and each well was added with 2 mL of the complete medium (DMEM, Gibco BRL). When about 80% of the bottom of the flask was covered with cells, PM2.5 (50 μg/mL) contamination was conducted, and the cells were divided into miR-217-5p NC group, miR-217-5p mimic group, miR-217-5p NC+PM2.5 group and miR-217-5p mimic+PM2.5 group, and all experiments were repeated 3 times.

### Detection of content of serum inflammatory factors by enzyme-linked immunosorbent assay (ELISA)

The whole blood was centrifuged at 3500 r/min and 4° C for 15 min, and the serum was obtained. The levels of serum inflammatory factors tumor, necrosis factor-α (TNF-α) and interleukin-10 (IL-10), were detected according to the instructions of the ELISA kit (Beijing 4A Biotech Co., Ltd).

### Hematoxylin-eosin (HE) staining

The thoracic cavity of mice was cut open along the anterior midline, the blood vessels and bronchi were cut off through the lateral end of the lung hilus closely along the lung lobe, and the surrounding connective tissues were bluntly separated. Fresh lung tissues were harvested from the middle lobe of the right lung, and rinsed with pre-cooled normal saline, and the excess water was absorbed dry using the filter paper. Then the lung tissues were fixed in 4% paraformaldehyde for 24 h, dehydrated with gradient ethanol, transparentized with xylene, embedded and sectioned. The sections were baked at 65° C for 2 h, subjected to deparaffinization and hydration with ethanol, stained with hematoxylin dye for 5 min, and washed with water, followed by color separation with 1% hydrochloric acid alcohol for 2 s. After the sections were returned to blue with 0.5% ammonia water, they were stained with eosin for 30 s, washed with water, dehydrated with ethanol, transparentized with xylene and sealed with neutral balsam. Finally, the morphology of the lung tissues was observed under an optical microscope.

### Dihydroethidium (DHE) staining

The lung tissues were sliced into 10 μm-thick sections using a freezing microtome and stuck on a cationic adhesion slide. Then DHE solution at a concentration of 1 μmol/L was dripped on the tissue section, followed by incubation at 37° C for 20 min in the dark. After washing twice with 0.01 mol/L phosphate-buffered saline (PBS) (5 min/time), the section was mounted, and the red fluorescence intensity was observed under a fluorescence microscope.

### Immunofluorescence

The cells were inoculated into a 12-well plate (1×10^5^ cells/well). After 24 h, the cells were washed with PBS for 3 times, fixed with 4% paraformaldehyde for 30 min, and washed again with PBS for 3 times (5 min/time), permeabilized with 0.1% TritonX-100 at room temperature for 25 min, and washed with PBS for 3 times (3 min/time), and sealed with 3% BSA at room temperature for 30 min, followed by incubation with sufficient CD68 primary antibodies/macrophage markers (diluted at 1:200) at 4° C overnight. Then fluorescent secondary antibodies were added for incubation at 20-37° C for 1 h in the dark, and the cells were washed with PBS with Tween 20 (PBST) for 3 times (3 min/time) and incubated with DAPI for 3-5 min in the dark, followed by nuclear staining. The samples were washed with PBST for 4 times (5 min/time), and observed and photographed under the fluorescence microscope finally.

### Western blotting

The RAW246.7 cells and mouse lung tissues were harvested, from which the total protein was extracted, and its concentration was detected by BCA method. The extracted protein was mixed with 4× loading buffer, boiled on a metal bath at 100° C for 10 min, and subjected to SDS-PAGE. Then the protein was transferred onto a nitrocellulose membrane, incubated with the phosphorylated (p)-STAT1, total (t)-STAT1, TNF-α, IFN-γ, p47, gp91, NADPH oxidase 4 (NOX4), these primary antibodies were purchased form Abcam and diluted 1:1000 in western blot experiments, and β-actin (1:3000 dilution) primary antibody diluent at 4° C overnight, and incubated with AlexaFluor488-labeled goat anti-rabbit IgG secondary antibody (Abcam, 1:2000 dilution) at room temperature for 2 h, followed by exposure and development by the ECL solution. Finally, the corresponding gray value was measured using ImageJ software.

### Statistical processing

The DEseq2 and ggpubr packages of R software (v3.6.1) were used for bioinformatics analysis. DEGs were analyzed by Wald test, and cytokines were compared between two groups by rank sum test. SPSS 23.0 software was used for statistical analysis of other indexes. The counting data were tested by Shapiro-Wilk test to determine whether the normal distribution was met, and when the P> 0.05, the data conformed to the normal distribution. Continuous variables with normal distribution were presents as mean ± standard deviation $\left(\bar{\chi }\pm \text{s}\right)$. Mean of two continuous normally distributed variables were compared by two independent sample T test. One-way analysis of variance was adopted for comparison between two groups, and LSD test for pairwise comparison. *p*<0.05 was considered to be statistically significant.
